# Cysteine-Rich Whey Protein Isolate (CR-WPI) Ameliorates Erectile Dysfunction by Diminishing Oxidative Stress via DDAH/ADMA/NOS Pathway

**DOI:** 10.1155/2022/8151917

**Published:** 2022-03-21

**Authors:** Kefan Li, Aiyun Zhu, Jimmy Gutman, Qiang Fu, Shuai Liu

**Affiliations:** ^1^Department of Urology, Shandong Provincial Hospital, Cheeloo College of Medicine, Shandong University, Jinan, Shandong 250021, China; ^2^Department of Urology, Shandong Provincial Hospital Affiliated to Shandong First Medical University, Jinan, Shandong 250021, China; ^3^Mcgill University, Canada

## Abstract

Nitric oxide synthase- (NOS-) dependent endothelial dysfunction induced by oxidative stress (OS) is assumed to play a pivotal role in the pathogenesis and progression of diabetes mellitus-related erectile dysfunction (DMED). Cysteine-rich whey protein isolate (CR-WPI) is a widely used protein supplement and has been confirmed to reduce reactive oxygen species (ROS) by increasing cellular antioxidant glutathione (GSH). However, it is currently unknown whether CR-WPI elicits therapeutic effects in DMED. Here, we provide diabetic rats with CR-WPI to determine its effect on DMED and the underlying mechanisms. The results suggest that CR-WPI supplementation increased GSH biosynthesis and reduced ROS content and simultaneously upregulated the dimethylarginine dimethylaminohydrolase (DDAH)/asymmetrical dimethylarginine (ADMA)/nitric oxide synthase (NOS) metabolic pathway. Evaluation of intracavernous pressure (ICP) also showed an improvement of penile erectile function in CR-WPI-treated rats. The results of the vitro cell culture showed that glutathione pretreatment protected corpus cavernosum smooth muscle cells (CCSMC) from H_2_O_2_-induced apoptosis by decreasing Caspase 9 and Caspase 3 expressions. These results augur well for the potential therapeutic application of dietary CR-WPI supplementation for treating diabetic erectile dysfunction.

## 1. Introduction

Diabetes mellitus (DM) is one of the most common risk factors for erectile dysfunction (ED); patients with diabetes are three times more likely to develop erectile dysfunction than those without diabetes [1, 2]. To date, the mechanism of DMED has not yet been fully elucidated. Mounting evidence shows that nitric oxide synthase- (NOS-) dependent endothelial dysfunction caused by oxidative stress plays a major role in the formation and progression of DMED [3, 4]. The phosphodiesterase-5 (PDE5) inhibitor sildenafil has been shown to protect NOS-dependent endothelial function from oxidative stress (OS) [5]. However, some patients with DMED tend to be refractory to PDE5 inhibitors, which are currently the first-line treatment drugs for ED [6]. It is necessary to explore novel therapies for the treatment of DMED. Previous studies have shown that antioxidants can reduce the production of ROS and improve endothelial function in diabetic-related ED, indicating that antioxidant therapy plays an important role in ameliorating DMED [7, 8]. Antioxidant supplementation appears to be a promising strategy for the treatment of diabetic erectile dysfunction.

Evidence from previous studies indicates that OS-induced nitric oxide- (NO-) mediated endothelial dysfunction is a key process in DMED [9]. NO is synthesized from L-arginine by NOS, which plays a fundamental role in cavernous smooth muscle relaxation and penile erection [10]. Asymmetrical dimethylarginine (ADMA) is an endogenous L-arginine analog that inhibits the synthesis of NO by competitively binding to the active site of NOS. ADMA is synthesized during protein arginine residue methylation by protein arginine methyltransferase 1 (PRMT1) and degraded by dimethylarginine dimethylaminohydrolase (DDAH) [11]. Elevated plasma ADMA has been shown to be related to impaired endothelial L-arginine/NO pathway and endothelium-dependent vasodilation dysfunction in diabetes mellitus [12]. Our previous studies have demonstrated that probucol improves erectile function by reducing OS by increasing DDAH levels and reducing ADMA contents [13]. Furthermore, the improvement in erectile function was also observed in age-ED rats through the regulation of the DDAH/ADMA/NOS metabolism pathway by reducing oxidative damage [14]. Furthermore, OS-induced apoptosis leads to a reduction in endothelial and smooth muscle cell content, which affects penile hemodynamics and erectile response [15]. Therefore, antioxidant therapy and regulation of the DDAH/ADMA/NOS pathway are potentially effective therapeutic approaches for treating diabetic ED.

Cysteine-rich whey protein isolate (CR-WPI) contains more than 90% proteins including serum albumin, alpha-lactalbumin, lactoferrin, and immunoglobulin. CR-WPI provides the amino acid precursor cysteine necessary for the synthesis of GSH, which is an endogenous antioxidant that plays a key role in the determination of the cellular redox state [16]. Whey protein has been confirmed to improve diabetes by increasing the endogenous synthesis of GSH [17]. The antioxidant effect of WPI has also been shown in many studies to treat diabetes and its complications [18]. Additionally, the CR-WPI has been demonstrated to improve clinical outcomes and GSH levels in a number of other human pathologies including cancer and hepatitis [19, 20]. However, no existing studies have reported the effect of CR-WPI on DMED.

Based on the important role of OS in the pathogenesis of DMED, we set forth to provide diabetic rats with a cysteine-rich whey protein isolate to determine its therapeutic effect on the DMED and further explore the underlying mechanism.

## 2. Materials and Methods

### 2.1. Experimental Animals

All experimental procedures for the handling of the animals were approved and assisted by the Animal Care and Use Committee of Shandong University (No. 2021-491, Jinan, China). Sixty-four 8-week-old male Sprague-Dawley rats weighing 230–250 g were provided by the Shandong University Laboratory Animal Center. The rats were housed individually in a specific pathogen-free environment with an ambient temperature of 22-24°C. All animals adapted to 12 h light-dark cycles for a week with unlimited access to food and water. To induce diabetes mellitus, forty-eight rats after 12 h fasting received a single intraperitoneal injection of 60 mg kg^−1^ streptozotocin (STZ, Sigma-Aldrich, St. Louis, MO). The other sixteen rats were injected with an equal volume of citrate-buffered saline and used as a sham group. After monitoring tail-vein blood glucose levels every 24 h using an Accusure 580 (Yuwell, China) for 72 h, animals with blood glucose levels that consistently exceeded 16.7 mmol l^−1^ were considered diabetic animals. After feeding for 8 weeks, DM rats received a subcutaneous injection of apomorphine (APO, 100 mg/kg, Sigma-Aldrich Chemical Co., St. Louis, MO, USA). After 30 min, erectile function was evaluated through observing penile hyperemia, length, and circumference of the rats. 100% (48/48) of the STZ rats that failed to achieve normal erections qualified as ED based on the APO test. These 48 DMED rats were randomly divided into six groups: the diabetic control group received an equal volume of sterile water for 30 or 60 days by oral gavage, and the experimental groups were supplemented with CR-WPI (ABD Bioactives, MarryHealth Enterprises North America Inc., Canada) dissolved in sterile water at doses of 100 mg kg^−1^ or 300 mg kg^−1^ once a day for 30 or 60 days by oral gavage. The rats in the sham group also received equivalent sterile water by oral gavage for 30 or 60 days. The blood glucose and body weight of each rats were recorded before and after CR-WPI treatment. See the Supplementary Materials for detailed results (Table S1).

### 2.2. Isolation and Treatment of Corpus Cavernosum Smooth Muscle Cells (CCSMCs)

The CCSMCs were isolated from the same batch of normal 10-week-old male Sprague-Dawley rat penises following the standard protocols described by previous studies [21]. The CCSMCs were then cultured in Dulbecco's modified eagle medium (DMEM) supplemented with 10% PBS, 100 U/ml penicillin, and 100 mg/ml streptomycin at 37°C in a humidified atmosphere of 5% CO_2_. Improved tissue slice inoculation and trypsin digestion were used for the primary culture. After 3 passages, cell identification was carried out with *α*-smooth muscle actin (*α*-SMA; Abcam, USA) by immunofluorescence. CCSMCs in the fourth passage were incubated with or without GSH (5 mM or 10 mM) for 6 h, and then, H_2_O_2_ was added to CCSMCs to induce apoptosis for 18 h. In contrast, CCSMCs received an equal volume of PBS as a control. The cells were washed twice with PBS and stained with Annexin V-propidium iodide double staining. To evaluate the effect of GSH on CCSMCs apoptosis, we assessed the viability of CCSMCs with or without GSH-pretreated after H_2_O_2_ treatment by flow cytometry (Becton, Dickinson Franklin, Lakes, NJ, USA). The intracellular Caspase 9 and Caspase 3 protein expressions were detected by western blot assay to assess the levels of apoptosis.

### 2.3. Evaluation of Erectile Function

After treatment for 30 or 60 days, we measured the maximum intracavernous pressure (Max ICP) and recorded the ratio of Max ICP/mean systemic arterial pressure (MAP) to assess erectile function. The rats were placed in the face-up position after being anesthetized with sodium pentobarbital (5%, intraperitoneal). The left carotid artery was cannulated using a PE-50 tube (Becton Dickinson & Co., Sparks, MD) to record systemic arterial blood pressure. For the measurement of ICP, a 23-gauge needle filled with heparinized saline solution (250 U/ml) was implanted into the same side of the corpus cavernosum. After isolating the cavernous nerve (CN), we used an electrode (15 Hz; pulse width 5 ms; 5 V; interval time 4 min) to stimulate the CN for the 60 s. Variations of ICP and MAP were recorded using a pressure transducer system PowerLab 26T (ADInstruments, Sydney, Australia) and analyzed by Labchart 8 software (ADInstruments, Sydney, Australia). A previous study revealed that the level of phosphorylated eNOS remained increased 10 minutes following cavernous nerve stimulation (NS) [22]. Similarly, nerve stimulation also likely affects ROS and GSH levels. To avoid these limitations, we detected the levels of eNOS, GSH, and ROS 30 min before and after NS. See the Supplementary Materials for detailed results (Figure S1).

### 2.4. Reactive Oxygen Species (ROS) Assay

Euthanasia was performed 30 min after evaluation of erectile function, and the skin covering the rat penis was incised to expose bilateral penile crura by removing part of the ischiocavernosus muscle and fascia. Then, os penis within the glans was removed. The penises were randomly divided into three sections with similar size and morphology perpendicularly to the long axis. One section of tissue was used for determining the levels of ROS, GSH, and ADMA in penile tissue. Another section of tissue was used for western blot assay. The final section of tissue was used for immunofluorescence staining. For ROS assay, penile tissues were made into frozen sections of 10 mm thick. Briefly, frozen penile sections were incubated with 1 *μ*mol/l DHE (Sigma Chemical Co., USA) under a humidified chamber for 30 min at room temperature, protected from light. ROS-induced fluorescence was detected using an LSM 510 laser scanning confocal microscope (ZEISS, German) with an excitation wavelength of 488 nm and an emission wavelength of 610 nm. The number of nuclei labeled by DHE was analyzed using Quantity One software (Bio-Rad, USA). ROS levels in penile tissues were measured by chemiluminescence using a LumiMax detection kit (Stratagene, La Jolla, CA) [23]. The probe luminol is oxidized by reactive oxygen species that include superoxide anion, hydrogen peroxide, and other free radicals in the reaction that emits photons of light, which are detected with a standard luminometer. The values were standardized to the content of protein and expressed as U/mg protein.

### 2.5. ELISA

After euthanization, penile tissue was collected as previously described. The penile tissue was placed in an ice bath, then washed twice with saline, dried with neutral filter paper, and transferred to a homogenizer. Subsequently, 10% tissue homogenates were made with 0.25 mol/l sucrose and 0.01 mol/l Tris buffer and were centrifuged (4°C) for 10 min at 8000 rpm. The supernatants were collected and prepared for further assay. The GSH content in penile tissue was determined using Ellman's reagent using a glutathione assay kit (Cat # BC1175, Solarbio, Beijing, China). The compounds formed between GSH and 5,5′-dithiobis-2-nitrobenoic acid (DTNB) were measured spectrophotometrically at 412 nm. ADMA concentration in penile tissue was determined using a commercial ADMA ELISA kit (Bio-Swamp Immunoassay R&D Center, Shanghai, China).

### 2.6. Western Blot Assay

The minced penile tissues were homogenized as previously described [24]. Penile tissues and CCSMCs were lysed in ice cold RIPA lysis buffer (Thermo Fisher Scientific, Waltham, MA, USA) containing 1% EDTA and a protease inhibitor cocktail. The BCA assay was used to determine protein concentrations of tissue and cell lysates. 20 *μ*g of protein samples was resolved on SDS–polyacrylamide gel and transferred to a polyvinylidene difluoride membrane (Millipore Corp., Bedford, MA, USA). The membrane was blocked with 5% skim milk for 1 h and subsequently incubated at 4°C overnight with primary antibodies against *β*-actin (1 : 1000; sc1616, Santa Cruz Biotechnology, Santa Cruz, CA, USA), *α*-SMA (1 : 1000; AF1032, Affinity Biosciences), eNOS (1 : 1000; AF0096, Affinity Biosciences), phosphorylated eNOS (Ser 1177) (p-eNOS; 1 : 500; sc81510, Santa Cruz Biotechnology, Santa Cruz, CA, USA), nNOS (1 : 600, SC5302, Santa Cruz Biotechnology, Santa Cruz, CA, USA), DDAH1 (1 : 1000; ab2231, Abcam, Cambridge, UK), DDAH2 (1 : 500; ab184166, Abcam, Cambridge, UK), Caspase 3 (D3R6Y) (1 : 1000; CST14220, Cell Signaling Technology), and Caspase 9 (1 : 2000; ab202068, Abcam, Cambridge, UK). According to the manufacturer's instructions, the horseradish peroxidase- (HRP-) labeled secondary antibody was diluted in an appropriate ratio. The membranes were washed three times in Tris-buffered saline containing 0.1% Tween 20 (TBST) and incubated with secondary antibodies at room temperature for 1 h. After rinsing three times for 10 min with TBST, the blot was visualized by LAS3000 Image Analyzer (Fujifilm, Tokyo, Japan) and analyzed using Multi Gauge software (Fujifilm, Tokyo, Japan).

### 2.7. Immunofluorescence Staining

The penile tissues were soaked in 4% paraformaldehyde for fixation and then transferred to 30% sucrose in PBS for dehydration overnight. The samples were embedded and cut into 5 *μ*m sections before mounting on glass slides. After immersion and blocking for 1 h, the slides were incubated with primary antibodies against *α*-smooth muscle actin (*α*-SMA, 1 : 1000; Abcam) and endothelial nitric oxide synthase (eNOS; 1 : 50; Abcam). Next, the slides were washed and incubated with Alexa Fluor-594-conjugated secondary antibodies (Invitrogen, Carlsbad, CA, USA). The nuclei were stained with DAPI reagent (Thermo Fisher, MA, USA) for 5 min. Finally, the slides were examined under a fluorescence microscope (Leica, Heidelberg, Germany).

### 2.8. Masson's Trichrome Staining

Masson's trichrome staining was performed to access the smooth muscle cell and collagen fibril expression in the corpus cavernosum. A Masson's trichrome stain kit (Dako Sciences, Glostrup, Denmark) was utilized. Briefly, collagenous fibers stained blue, elastic fibers stained brown, muscle fibers dyed red, and nuclei counterstained dark blue. Smooth muscle to collagen ratio was evaluated using Image-Pro Plus 5.0 software (Media Cybernetics, Inc., Bethesda, MD, USA).

### 2.9. Statistical Analysis

All experiments were repeated at least three times, and the whole data were expressed as means ± SD. Student's *t* test was performed to assess the changes in blood glucose and body weight before and after CR-WPI treatment. Other differences between multiple groups were analyzed using a one-way analysis of variance (ANOVA) and the Bonferroni multiple comparison posttest. All statistical analyzes were processed with GraphPad software (GraphPad Prism 8.0, La Jolla, CA) and SPSS 26.0 statistical software (SPSS; Chicago, IL, USA). Differences showing statistical significance were taken as *P* < 0.05.

## 3. Results

### 3.1. Assessment of Erectile Responses

**Figure 1:**
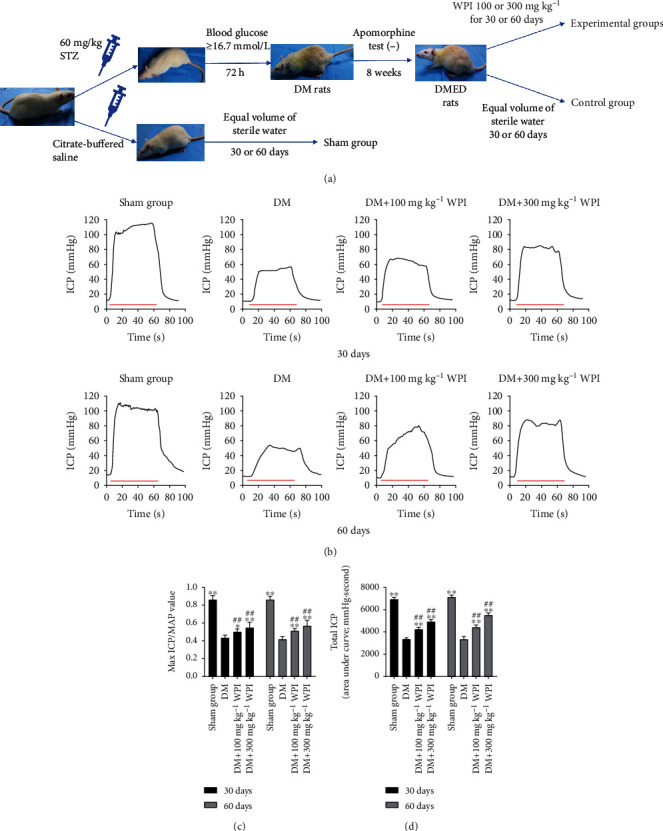
Flow diagram of the animal experiment and erectile responses of rats after treatment. (a) The process of the establishment of DMED rat model and treatment of the experimental animal. (b) CR-WPI supplementation improves the erectile function of DMED rats. The red lines denote electrical stimulation of the cavernous nerve for 60 s. (c) The ratio of maximum ICP to MAP derived from all eight groups (*n* = 8) is presented as bar graphs, and the data are shown as mean ± standard deviation. (d) The total ICP is obtained by calculating the area under curve, and the data are shown as mean ± standard deviation. ^∗^*P* < 0.05 and ^∗∗^*P* < 0.01 indicate a significant difference compared with the DM group. ^##^*P* < 0.01 indicates a significant difference compared with the sham group.

The ratio of maximum ICP/mean systemic arterial pressure (MAP) was used to assess erectile function. As shown in Figure 1, ICP and max ICP/MAP in diabetic rats were significantly lower than those in the sham group rats (*P* < 0.05). The CR-WPI treatment markedly improved ICP and max ICP/MAP in diabetic rats than untreated diabetic rats (*P* < 0.05), while the CR-WPI treatment group remained with lower ICP and max ICP/MAP than those in the sham group (*P* < 0.01).

### 3.2. Effects of CR-WPI on Oxidative Stress Levels

**Figure 2:**
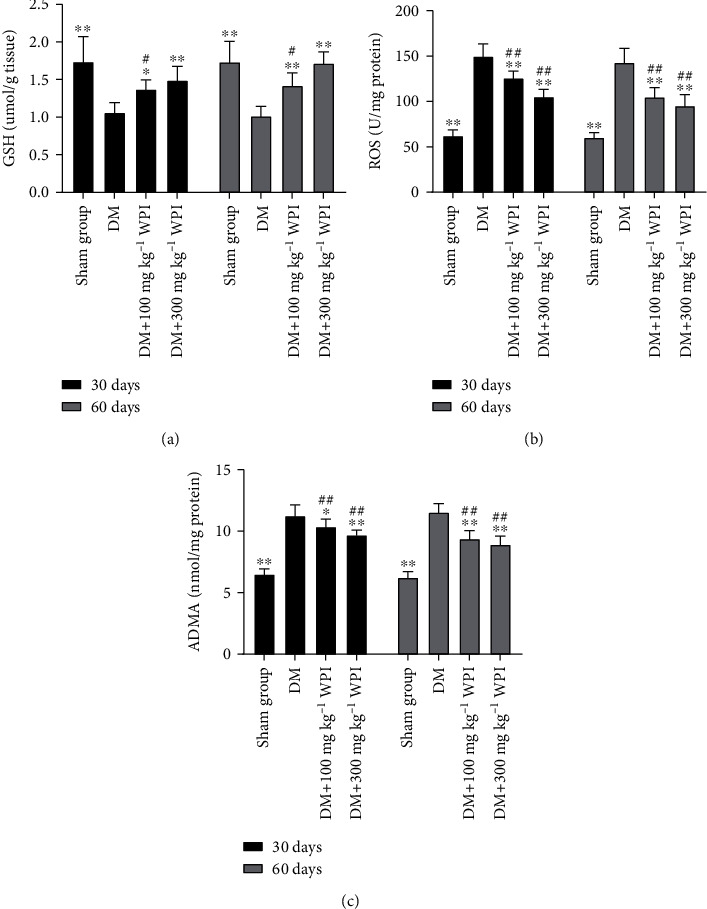
Effects of CR-WPI treatment on ROS, GSH, and ADMA levels in penile tissues after 30 days or 60 days treatment. (a) CR-WPI treatment increases GSH levels in the corpus cavernosum in a time and concentration-dependent manner. (b) CR-WPI treatment reduces ROS levels in diabetic rats. (c) CR-WPI supplementation decreases ADMA concentration of penile tissues in diabetic rats. ^∗^*P* < 0.05 and ^∗∗^*P* < 0.01 indicate a significant difference compared with the DM group. ^##^*P* < 0.01 indicates a significant difference compared with the sham group.

The contents of ROS and GSH were used to assess levels of OS. As demonstrated in Figures 2(a) and 2(b). Diabetic rats had significantly lower GSH content but significantly higher ROS levels compared to the sham group rats (*P* < 0.05). The rats in the CR-WPI-treated group remained with lower GSH contents but higher ROS levels compared with the sham group rats (*P* < 0.05). CR-WPI treatment increased GSH contents and decreased ROS levels compared with untreated diabetic rats (*P* < 0.05).

### 3.3. Effects of CR-WPI on DDAH/ADMA/NOS Metabolic Pathway

**Figure 3:**
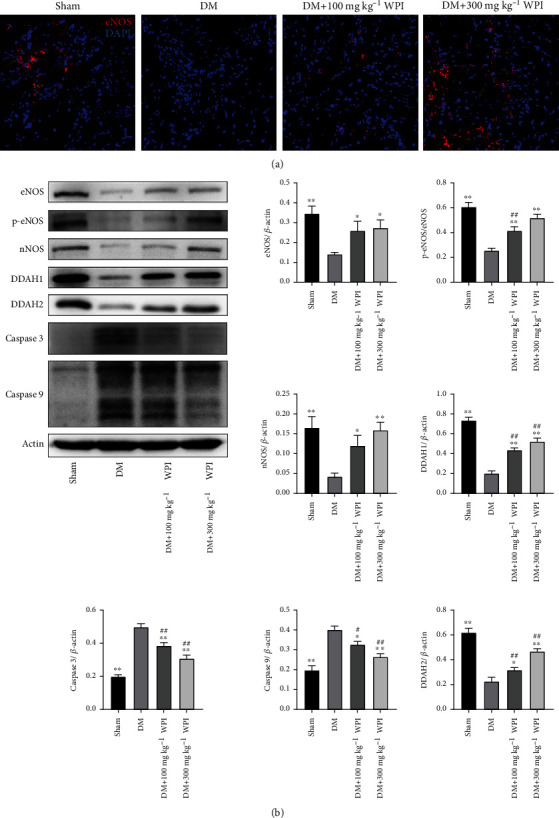
Changes of the DDAH/NOS pathway and apoptosis-related proteins in penile tissues after 100 mg kg^−1^ of and 300 mg kg^−1^ CR-WPI supplementation for 60 days. (a) Representative immunofluorescence staining of the eNOS-positive penile specimen (red) in the sham, DM, 100 mg kg^−1^ CR-WPI, and 300 mg kg^−1^ CR-WPI groups for 60 days. (b) The effects of CR-WPI on the protein expression of DDAH, NOS, Caspase 3, and Caspase 9 in diabetic rats. *β*-Actin was used as a loading control. ^∗^*P* < 0.01 and ^∗∗^*P* < 0.01 indicate a significant difference compared with the DM group. ^#^*P* < 0.05 and ^##^*P* < 0.01 indicate a significant difference compared with the sham group.

After treatment for 60 days, we analyzed the expression levels of the DDAH/ADMA/NOS metabolic pathway and apoptosis-related proteins by western blot assay and ELISA. As shown in Figure 2(c), the levels of ADMA, Caspase 3, and Caspase 9 were significantly increased in the diabetic control group compared with the sham group (*P* < 0.05). After 100 mg kg^−1^ or 300 mg kg^−1^ CR-WPI treatment, ADMA, Caspase 3, and Caspase 9 levels were dramatically reduced compared to the diabetic control group (*P* < 0.05). As shown in Figure 3, the levels of DDAH1, DDAH2, eNOS, and nNOS in the cavernous tissue were significantly lower in the diabetic control group than in the sham group (*P* < 0.05). The CR-WPI treatment group revealed elevated levels of DDAH1, DDAH2, eNOS, and nNOS compared to the diabetic control group (*P* < 0.05), although it remained lower than the sham group (*P* < 0.05).

### 3.4. Effects of CR-WPI on the Histological Structure of the Corpus Cavernosum

**Figure 4:**
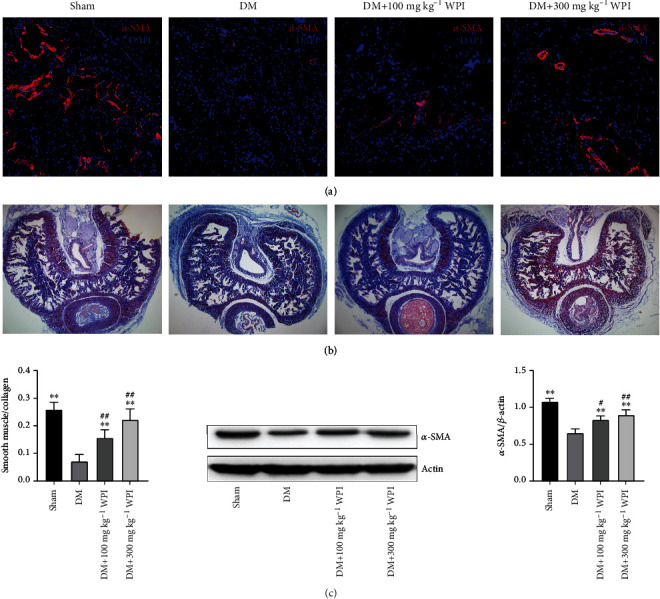
CR-WPI treatment for 60 days increases the smooth muscle content of the corpus cavernosum. (a) Representative immunofluorescence staining of the *α*-SMA-positive corpus cavernosum positive for *α*-SMA in the sham, DM, 100 mg kg^−1^ CR-WPI, and 300 mg kg^−1^ CR-WPI groups. (b) Masson's trichrome staining of penile tissue in four groups after different treatments. Smooth muscle and collagen in the corpus cavernosum are stained red and blue, respectively. (c) The effect of CR-WPI on the ratio of smooth muscle to collagen and protein expression of *α*-SMA (ratio to *β*-actin) in the corpus cavernosum. Bars denote the mean densitometry ratio between smooth muscle content and collagen content per field. ^∗∗^*P* < 0.01 compared to the DM group and ^#^*P* < 0.05 and ^##^*P* < 0.01 compared to the sham group.

We used Masson's trichrome staining, immunofluorescence staining of *α*-SMA, and western blot of *α*-SMA to determine histological changes of the corpus cavernosum after 60 days of treatment. As presented in Figure 4, the ratio of smooth muscle to collagen was lower in the diabetic control group and CR-WPI-treated group compared with the sham group (*P* < 0.05). After CR-WPI treatment, the ratio of smooth muscle to collagen was significantly higher compared with the diabetic control group (*P* < 0.05). CR-WPI treatment increased the content of smooth muscle in the corpus cavernosum compared to the diabetic control group (*P* < 0.05). The level of *α*-SMA detected by western blot assay was lower in the diabetic control group and the CR-WPI-treated group than in the sham group (*P* < 0.05). The CR-WPI treatment ameliorated the reduction of *α*-SMA level compared with the diabetic control group (*P* < 0.05).

### 3.5. Effects of CR-WPI on Apoptosis of CCSMCs

**Figure 5:**
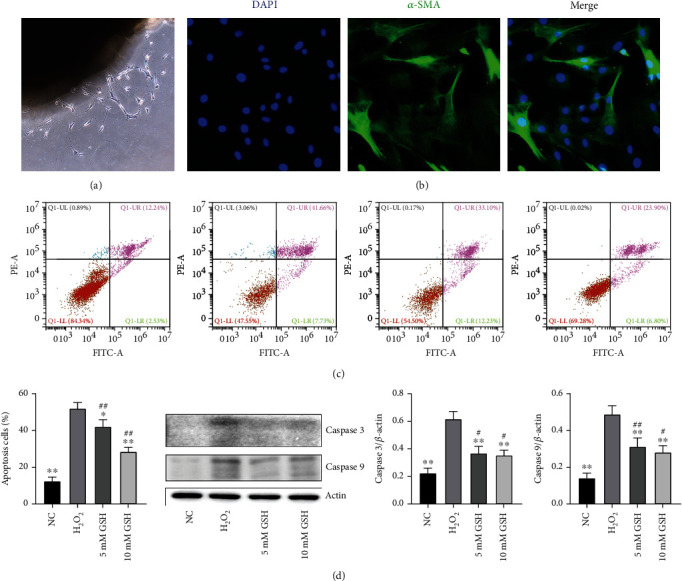
Effect of GSH on CCSMC apoptosis. (a) Primary CCSMCs emerging from the corpus cavernosum tissue after 3 days. (b) Immunofluorescence with anti-*α*-SMA antibody for CCSMC identification, ×40 amplification. (c) Cell viability was detected by flow cytometry in the normal control group treated with PBS (NC) and the H_2_O_2_-treated groups (pretreated with 0, 5, and 10 mM GSH). (d) The average apoptotic cell percentages and expression of Caspase 3 and Caspase 9 of CCSMCs in the NC group, H_2_O_2_ group, 5 mM GSH, and 10 mM GSH group. Data are expressed as mean ± SD, *n* = 3. ^∗^*P* < 0.05 and ^∗∗^*P* < 0.01 indicate a significant difference compared with the H_2_O_2_ group. ^#^*P* < 0.05 and ^##^*P* < 0.01 indicate a significant difference compared with the NC group.

The cells were stained with Annexin V-propidium iodide double staining and counted with a flow cytometer (Figure 5(c)). When the cells were incubated with 5 mM or 10 mM GSH, the proportion of apoptotic cells was42.09 ± 3.01 or 28.49 ± 1.64 (Figure 5(d)), while when the cells were incubated in the absence of GSH, the proportion of apoptotic cells was 52.08 ± 4.45. The protein expression levels of Caspase 3 and cleaved Caspase 3 in CCSMCs detected by western blot are shown in Figure 5(d). The proapoptotic Caspase 3 and cleaved Caspase 3 expressions were significantly increased in the H_2_O_2_-treated CCSMCs than the PBS control group (*P* < 0.05). GSH pretreatment significantly decreased Caspase 3 and cleaved Caspase 3 expressions compared to CCSMC pretreated with PBS (*P* < 0.05).

## 4. Discussion

Currently, DMED remains a treatment dilemma due to its low sensitivity to first-line treatment therapy with PDE-5 inhibitors [25]. Oxidative damage induced by DM is the critical mechanism in DMED [26]. OS could alter vascular endothelial diastolic function, reduce blood flow, and destroy the tissue structure of the corpus cavernosum [27]. As shown in previous studies [7, 8], antioxidant therapy appeared to be a promising protocol for protecting endothelium-dependent vasodilation in DMED. However, the mechanism of improvement in endothelial diastolic function is not yet fully clarified. Our previous study confirmed that antioxidant probucol improved erectile function in diabetic rats by reducing OS [13]. However, the therapeutic value of probucol is limited by its side effects, such as gastrointestinal irritation, prolongation of the QT interval, and possible arrhythmias [28]. Probucol seems to be not cost-effective for the treatment of DMED. Therefore, it is necessary to find a safer and more effective therapy for the treatment of DMED.

**Figure 6:**
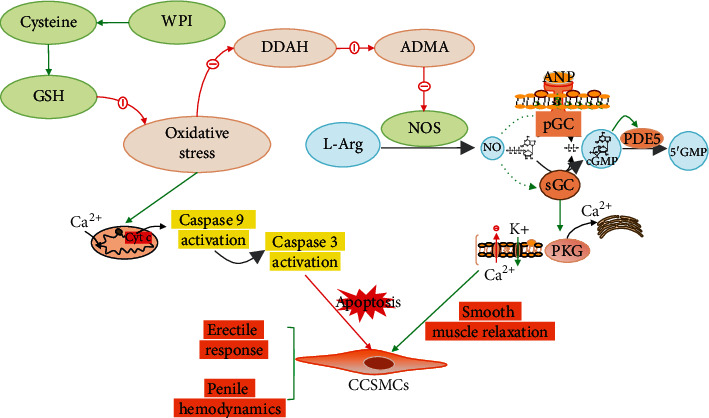
Diagram of the molecular mechanism of how CR-WPI affects erectile function. DDAH: dimethylarginine dimethylaminohydrolase; ADMA: asymmetric dimethylaminohydrolase; L-Arg: L-arginine; GC: guanylyl cyclase; PDE5: phosphodiesterase-5; PKG: cGMP-dependent protein kinase.

Dietary supplementation to increase intracellular GSH content has been proven to be an effective and reliable way to treat diabetes and its complications in rodents [29]. These supplements include dietary GSH, GSH precursor N-acetylcysteine (NAC), and cysteine. However, in comparison to rodents, oral GSH in humans is generally considered to have little effect on tissue glutathione [30]. The susceptible metabolism of cysteine in the gastrointestinal tract and the relatively low stability and bioavailability of oral GSH have limited their efficacy and further development [31]. The cysteine-rich whey protein isolate (CR-WPI) contains abundant cystine (two cysteine molecules connected by an intermolecular disulfide bond) and has been widely used for its safety and efficacy [32]. Compared with other dietary GSH supplements or GSH precursors, CR-WPI is more stable and with higher bioavailability because the cystine is resistant to pepsin and trypsin [33]. In this study, we tried to investigate whether CR-WPI supplementation ameliorates diabetic erectile dysfunction. Our results demonstrated that CR-WPI supplementation improved diabetic erectile function in a streptozotocin-induced diabetic rat model. What, then, is the mechanism underlying? We focus on the NOS metabolic pathway and thought it could be achieved by increasing endogenous GSH content to reduce OS, which regulates the metabolic pathway DDAH/ADMA/NOS and inhibits apoptosis of CCSMC (Figure 6).

The present study determined the content of GSH and ROS to assess the level of OS in corpus cavernosum tissue. The diabetic rats showed a decreased GSH content and raised ROS level compared with normal rats, indicating an increased OS level in DMED. The anomalies in DMED rats could be attenuated by CR-WPI supplementation, which suggested that CR-WPI treatment reduced OS levels by decreasing ROS production and restoring GSH content in the corpus cavernosum. The result was in agreement with the previous study that CR-WPI supplementation increased the intracellular GSH levels and augmented antioxidant defenses [34]. Tagliabue et al. compared diabetic and nondiabetic erectile dysfunction patients and found lower GSH levels in DMED patients; moreover, GSH depletion could bring about a reduction of NO synthesis, followed by a vasodilation dysfunction in the corpora cavernosa [35]. It is well known that NO is synthesized by NOS in the presence of L-arginine, which is regulated by the DDAH/ADMA/NOS pathway. OS has been proven to inactivate DDAH and increase ADMA concentration and contribute to the reduction of NO production [36]. A previous study has shown that the promotion of GSH synthesis restores DDAH activity and reduces ADMA contents in young spontaneously hypertensive rats [37]. Another study revealed that dairy milk proteins such as whey protein attenuates hyperglycemia-induced impairment in endothelial function by diminishing OS to decrease ADMA levels and increase NO bioavailability [38]. Additionally, DDAH is sensitive to OS due to the existence of critical sulfhydryls at the active site of the enzyme; an early study showed that GSH participated in NO synthesis as a cofactor [39, 40]. These studies are consistent with the findings in our research that CR-WPI supplementation restored the contents of GSH and reduced ROS production and then upregulated the DDAH/ADMA/NOS pathway.

The present study also evaluated the apoptosis of the corpus cavernosum in vitro culture of CCSMC. Previous experiments have shown that ROS can oxidize cellular GSH or accelerate its efflux from the cell, leading to the loss of intracellular redox homeostasis and activation of the apoptotic caspase cascade reaction [41]. OS promotes mitochondrial depolarization, overloads Ca^2+^ entry, and then induces the release of cytochrome C into the cytosol, the sequence of events that trigger caspase-dependent apoptosis [42]. Caspase 3 is a critical protease in apoptosis and is considered the common pathway of the caspase cascade [43]. In our research, GSH pretreatment reduced the Caspase 9 and Caspase 3 expressions in CCSMCs induced by hydrogen peroxide compared with PBS-pretreated CCSMCs. Another study also validated our results that GSH depletion related to apoptosis and pretreatment of cells with GSH reduced ROS production and apoptosis [44].

Furthermore, the structure of the corpora cavernosa was analyzed by Masson's trichrome staining and immunofluorescence staining with *α*-SMA. Prior studies have demonstrated that cavernosal smooth muscle and *α*-SMA contents were decreased due to the deleterious effect of oxidative stress in diabetes-associated ED [26]. The current study indicated that supplementation with CR-WPI increased the expression of *α*-SMA and the ratio of smooth muscle to collagen in the corpora cavernosa of DMED rats. This could be related to attenuation of OS and reduction of CCSMC apoptosis. However, the specific mechanism still requires further elucidation.

Although our study proves the therapeutic effects of CR-WPI on erectile dysfunction of DM rats in vivo and in vitro, some limitations remain. The main limitation of this study is only animal experiments have been carried out, and it remains unknown whether it elicits therapeutic effects in DMED patients. Another limitation is that the animal model used in this study is similar to type 1 diabetes, while type 2 diabetes accounts for the majority of diabetic patients. Therefore, the generalizability of the results may be limited.

## 5. Conclusions

In this research, we demonstrate that the supplementation with cysteine-rich whey protein isolate (CR-WPI) promotes diabetic erectile function by increasing endogenous glutathione levels to attenuate the deleterious effects of oxidative stress. Meanwhile, it regulates the DDAH/ADMA/NOS metabolic pathway and decreases the apoptosis of CCSMC. Additionally, recovery of smooth muscles and improvement of architectural changes in the corpus cavernosum improve penile hemodynamic function for erection. All these results promise the future application of cysteine-rich whey protein isolates for the treatment of diabetic erectile dysfunction.

## Data Availability

All the data generated or analyzed during the present study are included in this article.

## Supplementary Materials

Supplementary Materials Body weight and blood glucose in the experimental animals.
