# Supplementary material for: Cysteine-Rich Whey Protein Isolate (CR-WPI) Ameliorates Erectile Dysfunction by Diminishing Oxidative Stress via DDAH/ADMA/NOS Pathway
Source: Oxid Med Cell Longev. 2022 Mar 21;2022:8151917. doi: 10.1155/2022/8151917 (PMC8960025; doi:10.1155/2022/8151917)
Supplement: Supplementary Materials — Body weight and blood glucose in the experimental animals. [file 8151917.f1.docx]

|  |  | Sham group | DM group | DM+WPI 100 mg kg^-1^ group | DM+WPI 300 mg kg^-1^ group |
| --- | --- | --- | --- | --- | --- |
| Initial | Body weight (g) | 299.5 ± 15.95 | 283.9 ± 10.67 | 297.0 ± 14.41 | 297.8 ± 13.89 |
|  | Glucose (mmol/L) | 6.31 ± 0.48 | 21.14 ± 1.93 | 23.16 ± 2.26 | 22.36 ± 1.85 |
| 60 days | Body weight (g) | 517.2 ± 48.17 | 329.8 ± 20.98** | 376.4 ±10.98^#^ | 386.7 ± 11.90^#^ |
|  | Glucose (mmol/L) | 6.16 ± 0.51 | 21.04 ± 1.48** | 22.75 ± 1.63** | 21.31 ± 1.55** |

**Table S1** Comparisons of body weight and blood glucose in experimental animals

Data are expressed as mean ± standard deviation (n= 8 per group) from four groups: Sham group; DM, diabetes mellitus group; DM+WPI 100mg kg^-1^ group; DM+WPI 300mg kg^-1^ group. **P < 0.01 indicate significant difference compared with the sham group. ^#^P < 0.05 indicates significant difference compared with the DM group.

**Figure. S1** The levels of eNOS, GSH, and ROS in penile tissues 30 min before and after nerve stimulation (NS) of the cavernous nerve. The results show no significant differences 30 min before and after NS.

**
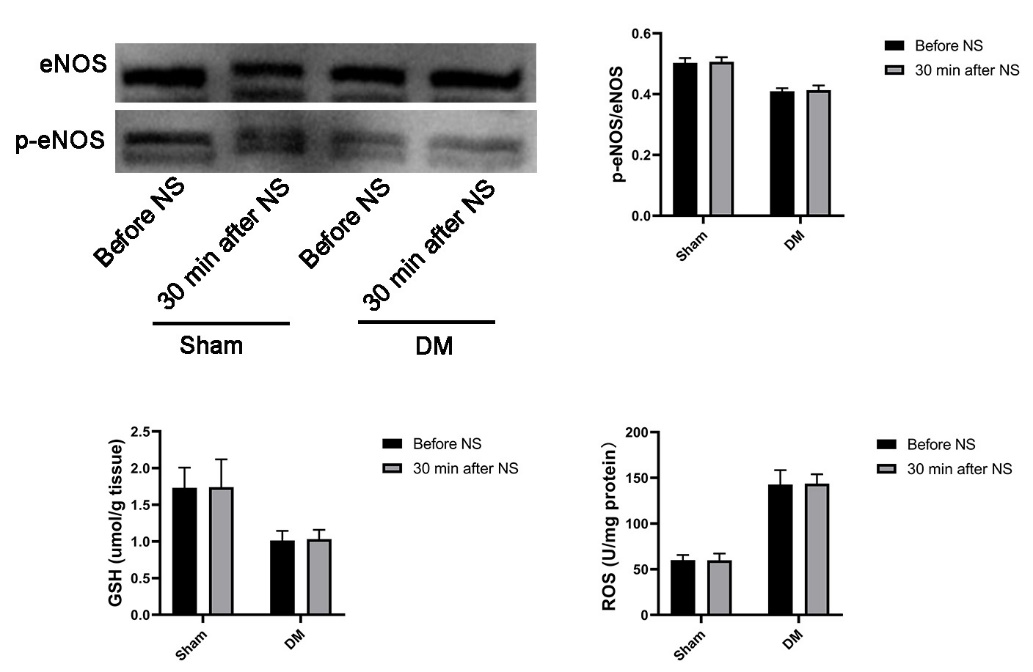
**
